# Returning incidentally discovered Hepatitis C RNA-seq results to COPDGene study participants

**DOI:** 10.1038/s41525-023-00379-4

**Published:** 2023-10-31

**Authors:** Edwin K. Silverman, Arthur Y. Kim, Barry J. Make, Elizabeth A. Regan, Jarrett D. Morrow, Craig P. Hersh, James O’Brien, James D. Crapo, Nadia N. Hansel, Gerard Criner, Eric L. Flenaugh, Douglas Conrad, Richard Casaburi, Russell P. Bowler, Nicola A. Hanania, R. Graham Barr, Surya P. Bhatt, Frank C. Sciurba, Antonio Anzueto, MeiLan K. Han, Charlene E. McEvoy, Alejandro P. Comellas, Dawn L. DeMeo, Richard Rosiello, Jeffrey L. Curtis, Tricia Uchida, Carla Wilson, P. Pearl O’Rourke

**Affiliations:** 1 Channing Division of Network Medicine, Department of Medicine, Brigham and Women’s Hospital, Harvard Medical School, Boston, MA USA; 2 Division of Pulmonary and Critical Care Medicine, Department of Medicine, Brigham and Women’s Hospital, Harvard Medical School, Boston, MA USA; 3 Division of Infectious Diseases, Department of Medicine, Massachusetts General Hospital, Harvard Medical School, Boston, MA USA; 4 Department of Medicine, National Jewish Health, Denver, CO USA; 5 Division of Pulmonary and Critical Care Medicine, School of Medicine, Johns Hopkins University, Baltimore, MD USA; 6 Department of Thoracic Medicine and Surgery, Temple University, Philadelphia, PA USA; 7 Pulmonary and Critical Care and Interventional Pulmonary Medicine, Morehouse School of Medicine, Atlanta, GA USA; 8 Department of Medicine, University of California San Diego, La Jolla, CA USA; 9 Rehabilitation Clinical Trials Center, Division of Respiratory and Critical Care Physiology and Medicine, Lundquist Institute for Biomedical Innovation at Harbor-UCLA Medical Center, Torrance, CA USA; 10 Section of Pulmonary and Critical Care Medicine, Baylor College of Medicine, Houston, TX USA; 11 Departments of Medicine and Epidemiology, Columbia University Medical Center, New York, NY USA; 12 Division of Pulmonary, Allergy, and Critical Care Medicine, University of Alabama at Birmingham, Birmingham, AL USA; 13 Division of Pulmonary, Allergy and Critical Care Medicine, Department of Medicine, University of Pittsburgh, Pittsburgh, PA USA; 14 Pulmonary and Critical Care, University of Texas Health, and South Texas Veterans Health Care System, San Antonio, TX USA; 15 Division of Pulmonary and Critical Care Medicine, Department of Medicine, University of Michigan, Ann Arbor, MI USA; 16 HealthPartners Institute, Bloomington, MN USA; 17 Division of Pulmonary, Critical Care and Occupational Medicine, University of Iowa Hospital and Clinics, Iowa City, IA USA; 18 Department of Pulmonary and Critical Care, Reliant Medical Group, Worcester, MA USA; 19 Medical Service, VA Ann Arbor Healthcare System, Ann Arbor, MI USA; 20 Research Informatics Services, National Jewish Health, Denver, CO USA; 21 Harvard Medical School (retired), Boston, MA USA

**Keywords:** Medical ethics, Hepatitis C

## Abstract

The consequences of returning infectious pathogen test results identified incidentally in research studies have not been well-studied. Concerns include identification of an important health issue for individuals, accuracy of research test results, public health impact, potential emotional distress for participants, and need for IRB permissions. Blood RNA-sequencing analysis for non-human RNA in 3984 participants from the COPDGene study identified 228 participants with evidence suggestive for hepatitis C virus (HCV) infection. We hypothesized that incidentally discovered HCV results could be effectively returned to COPDGene participants with attention to the identified concerns. In conjunction with a COPDGene Participant Advisory Panel, we developed and obtained IRB approval for a process of returning HCV research results and an HCV Follow-Up Study questionnaire to capture information about previous HCV diagnosis and treatment information and participant reactions to return of HCV results. During phone calls following the initial HCV notification letter, 84 of 124 participants who could be contacted (67.7%) volunteered that they had been previously diagnosed with HCV infection. Thirty-one of these 124 COPDGene participants were enrolled in the HCV Follow-Up Study. Five of the 31 HCV Follow-Up Study participants did not report a previous diagnosis of HCV. For four of these participants, subsequent clinical HCV testing confirmed HCV infection. Thus, 30/31 Follow-Up Study participants had confirmed HCV diagnoses, supporting the accuracy of the HCV research test results. However, the limited number of participants in the Follow-Up Study precludes an accurate assessment of the false-positive and false-negative rates of the research RNA sequencing evidence for HCV. Most HCV Follow-Up Study participants (29/31) were supportive of returning HCV research results, and most participants found the process for returning HCV results to be informative and not upsetting. Newly diagnosed participants were more likely to be pleased to learn about a potentially curable infection (*p* = 0.027) and showed a trend toward being more frightened by the potential health risks of HCV (*p* = 0.11). We conclude that HCV results identified incidentally during transcriptomic research studies can be successfully returned to research study participants with a carefully designed process.

## Introduction

The incidental discovery of potentially clinically important information during a research study raises serious ethical and legal issues. There is no consensus regarding whether to return these incidental results to study participants or the best approach for returning such results if returning results is pursued^1^. In contrast, the return of incidental genetic findings identified during clinical genome sequencing has become standard, and a panel of actionable genes has been selected by the American College of Medical Genetics and Genomics (ACMG)^2^. In response to the return of these actionable genetic variants in clinical settings, a number of recent research population studies, including “All of Us”^3^, plan return of these same variants as explicitly described in their informed consent. As demonstrated in a Pro-Con debate in the New England Journal of Medicine^4^, there is ongoing controversy regarding return of genetic results to research study participants who did not provide informed consent to receive those results. Many genetic epidemiologists do not return incidentally discovered genetic results to participants if return of results was not included in the informed consent, and they believe that genetic epidemiology studies should be viewed differently than clinical genetic testing due to the large sample sizes involved and lack of clinical relationships with study participants^5^.

The issue of how to handle incidental research findings is not limited to genetics. While there are studies in which non-genetic incidental findings have been returned, the question of whether or not to return a specific result remains a challenge. This challenge will likely increase as the amount of research generated data expands. Consider programs like the NHLBI’s Trans-Omics for Precision Medicine (TOPMed) that collects genetic, transcriptomic, metabolomic, proteomic, and epigenetic data. These comprehensive biological assessments will likely provide novel insights into disease pathogenesis, but also could be informative about existing clinical conditions, such as infectious diseases. Incidentally discovered findings from Omics data generation will likely provide increasing challenges to medical researchers.

The return of infectious pathogen test results has not been well-studied or effectively addressed. Although scant medical literature exists on return of viral results in research settings, blood banking organizations have long-standing experience with returning such results to potential blood donors. However, potential blood donors are informed before donation that they would be notified if a positive test is found for infections including hepatitis, human immunodeficiency virus (HIV), and syphilis (www.redcrossblood.org/donate-blood/manage-my-donations/rapidpass/what-you-must-know-before-blood-donation.html)^6^. Studies of blood donors positive for hepatitis C virus (HCV) in the United Kingdom showed high rates of satisfaction with the notification process^7^ but low rates of downstream follow-up^8^. To our knowledge, no study has examined the impact of incidental research viral result notification in the U.S. during the era of effective HCV therapies.

HCV is an RNA virus transmitted by sexual and blood-borne contact that causes acute infection within six months of exposure to the virus; over half of the individuals with acute infection develop chronic HCV infection. Chronic HCV infection is relatively common with major implications for both personal and public health; the estimated burden in the US is at least 2.4 million HCV subjects^9^. After a latent period lasting decades, chronic HCV can cause liver damage, cirrhosis, liver cancer, and death. Fortunately, HCV can now be treated effectively, thereby preventing onward transmission and downstream complications including death^10,11^. HCV treatment became far safer and more effective after the approval of direct-acting antivirals (DAAs) in December 2013. However, a variety of factors, including their cost, have limited broad application of these curative therapies^12^.

COPDGene, an NHLBI-funded observational longitudinal study focused on chronic obstructive pulmonary disease (COPD)^13^, obtained whole blood transcriptomics to investigate COPD pathobiology and heterogeneity. In subsequent analysis of these blood RNA-seq data for non-human transcripts^14^, 228 individuals with suggestive evidence for hepatitis C virus (HCV) were discovered in a research laboratory that was not governed by the Clinical Laboratory Improvements Amendments of 1988 (CLIA). These results were unanticipated, and they provided both an opportunity and a challenge for returning results with personal and public health implications to COPDGene study participants. Return of infectious disease results to research study participants could lead to improvements in their health as well as the health of their personal contacts, but if the results are not provided in an empathetic and informative manner, serious harm could be inflicted as well. We hypothesized that incidentally discovered HCV results could be effectively returned to COPDGene participants, with the potential to lead to clinically indicated health interventions.

## Results

### HCV return of results

**Fig. 1:**
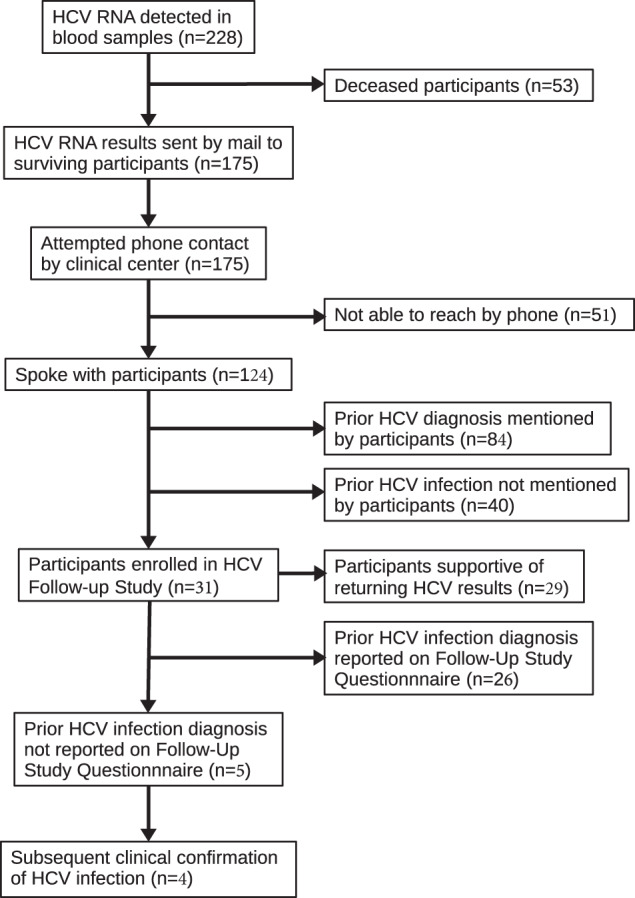
CONSORT Diagram for Return of HCV Results in COPDGene.

A multi-step approach to return HCV results to COPDGene participants was developed (see Table 1 and Methods). As shown in Fig. 1, efforts were made to contact 175 participants with evidence of HCV, who were not deceased. One hundred and twenty-four participants successfully completed initial phone calls, and 84 of these 124 participants (67.7%) spontaneously volunteered that they had been previously diagnosed with HCV.

**Table 1:** Procedure for returning incidental HCV results to COPDGene participants.

Procedural Step	Comments
1) Initial Letter to HCV-positive Participants (Based on RNA-seq)	Sent by certified mail from COPDGene Clinical Centers
2) Initial Phone Call to HCV-positive Participants	Trained Clinical Center staff confirmed receipt and understanding of initial HCV letter and assessed interest in receiving HCV Follow-Up Study Information. Recorded whether participant spontaneously volunteered that they had been previously diagnosed with HCV
3) Second Mailing with Follow-Up Study Information	Provided HCV Follow-up Study consent form and medical records form, if interested
4) Second Phone Call to Assess Interest in Follow-up Study Participation	Review of HCV Follow-up Study consent form and medical records form
5) Collection of Consent Form for Follow-up Study and Medical Records Release Form	
6) Letter to Participant’s Physician	
7) Completion of Follow-up Study Questionnaire	Phone interview three months after initial contact letter
8) Medical Records Collection	De-identified medical records transmitted to COPDGene Data Coordinating Center for central review of previous HCV diagnostic testing, liver evaluation, and HCV treatment

Thirty-one participants consented to participate and provided questionnaire data in the HCV Follow-up Study. While 26 of the 31 participants reported a previous diagnosis of HCV, five did not. For four of these five participants, subsequent clinical HCV testing confirmed HCV infection. One participant had negative clinical HCV testing; of interest, that participant was at the lower limit of HCV detection in their COPDGene research blood RNA-seq analysis.

Medical records were obtained for 14 COPDGene participants in the HCV Follow-up Study. As shown in Table 2, HCV diagnoses were confirmed for 13/14 participants, and the non-confirmed participant provided limited medical records and reported a previous HCV diagnosis on their HCV Follow-up Questionnaire. Eight of the 14 participants had medical record documentation of previous treatment with anti-viral agents for HCV.

**Table 2:** Medical records for 14 of 31 of COPDGene HCV follow-Up study participants.

Participant	Confirmed HCV Diagnosis?	HCV Testing Type	Liver Imaging and/or Liver Biopsy	HCV Treatment: Drug(s) Used
1	Yes	HCV Ab Reactive HCV RT-PCR	Not provided	Not provided
2	Yes	HCV RNA	Not provided	Not provided
3	Yes	HCV RNA 0 IU/ml (confirmed successful treatment)	Not provided	Not provided
4	Yes	HCV genotype	Liver biopsy with bridging fibrosis (score 3/6)	Pegylated IFN and Ribavirin
5	Yes	HCV genotype HCV Ab HCV RNA	Office note mentions “moderate fibrosis”	elbasvir/grazoprevir
6	Yes	HCV PCR	RUQ U/S: Fatty liver infiltration Abd CT: Normal liver Liver biopsy with bridging fibrosis (3/6)	Not provided
7	Yes	HCV genotype	RUQ U/S normal liver	sofosbuvir/velpatasvir; previously treated with IFN
8	Yes	HCV RNA	RUQ U/S normal Fibroscan with fibrosis stage F0-F1	sofosbuvir/velpatasvir
9	Yes	HCV Ab reactive	Not provided	Not provided
10	Yes	HCV RNA	Liver CT: Cirrhotic morphology; Abdominal MRI with cirrhosis	sofosbuvir/velpatasvir
11	Yes	HCV RNA HCV genotype	F2 fibrosis level	ledipasvir/sofosbuvir
12	Not mentioned in medical record, but previous diagnosis noted in Follow-up Questionnaire	No testing provided	RUQ U/S with hepatomegaly	Not provided
13	Yes	HCV genotype Negative HCV quantitative PCR after treatment	Not provided	IFN-based therapy then ledipasvir/sofosbuvir
14	Yes	HCV RNA HCV genotype	Liver Bx with chronic hepatitis (moderate to severe); mild fibrous portal expansion with bridging fibrosis	IFN-based therapy then simeprevir/sofosbuvir

*RUQ* Right upper quadrant, *Bx* Biopsy, *IFN* Interferon, *Ab* Antibody, *U/S* Ultrasound, *RT-PCR* Real-time polymerase chain reaction, *IU* International Units, *ml* milliliter, *Abd* Abdominal, *CT* Computed tomography.

### Participant perceptions about HCV return of results

**Fig. 2:**
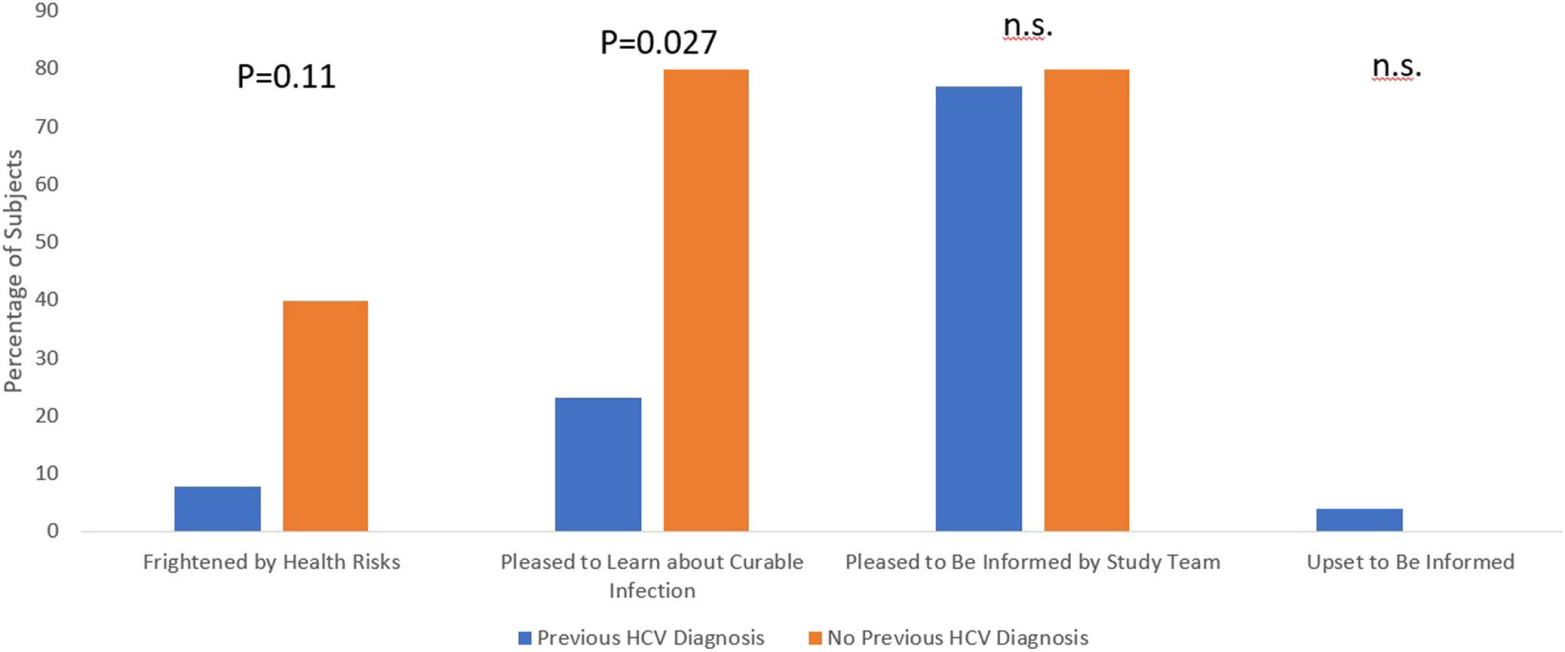
Reactions to being informed about possible HCV infection stratified by previous HCV Diagnosis. Subjects with (*n* = 26) and without (*n* = 5) a previous HCV diagnosis were compared using Fisher’s Exact Tests. n.s. not significant.

Based on the HCV Follow-up Study survey data, 93.5% of participants (29/31) were supportive of returning HCV research results to study participants. Regarding the initial contact letter, 17 participants found it to be Very Informative, 11 found it to be Informative, two found it to be Neutral, and one found it to be Confusing. Regarding the staff phone call to review the notification letter, responses included: 15 Very Informative, 10 Informative, four Neutral, and two Confusing. Participants were able to provide multiple responses regarding their emotional reactions to receiving the HCV information: 24 were pleased to be informed; ten were pleased to learn that HCV is a potentially curable infection; four were frightened by the health risks of HCV; and one was upset that the study team contacted them with a laboratory test result that they did not expect to receive. The participant who was upset to receive HCV information was previously diagnosed with HCV; one other participant indicated they were surprised and upset that someone other than their physician knew about their previously diagnosed HCV. The reactions of HCV Follow-Up Study participants, stratified by previous HCV diagnosis, are shown in Fig. 2. The small number of newly diagnosed participants (*n* = 5) were more likely to be pleased to learn about a potentially curable infection (*p* = 0.027) and showed a trend toward being more frightened by the potential health risks of HCV (*p* = 0.11).

Several participants asked their clinical center why the HCV results were only being reported in 2021. An additional letter describing the timeline of obtaining results was generated and IRB-approved. This letter clarified that, although COPDGene started in 2008, the HCV results were based on blood samples obtained at the five-year follow-up visits (performed between 2013 and 2017) and the assessment of non-human RNA sequences started at the end of 2019. Additional analyses were performed to verify the findings, and the HCV results were finalized at the end of 2020.

## Discussion

After incidentally discovering evidence for HCV based on non-CLIA, research RNA-sequencing in the COPDGene study, we developed a process for returning these HCV results to study participants that was approved by a COPDGene Participant Advisory Panel as well as by the Observational Safety and Monitoring Board (OSMB) and IRBs monitoring the COPDGene project. Based on the high rate of previously reported HCV infections spontaneously volunteered by participants at the initial phone call as well as the very high rates of either previously or newly diagnosed HCV among HCV Follow-up Study surveys and medical record reviews, we contend that the HCV diagnoses from this research-based RNA-sequencing are at least reasonably accurate. We identified four new diagnoses of HCV that were confirmed by subsequent clinical testing. One additional participant with a low copy number of HCV was not confirmed clinically.

Knowledge of infection is a prerequisite for receiving effective therapy. Clinical HCV testing typically involves a two-stage process which tests first for antibodies (anti-HCV) indicating exposure, then is followed by nucleic acid testing (NAT, also termed HCV RNA) indicating active or “current” infection. The U.S. Centers for Disease Control and Prevention (CDC) estimates that 1% of the U.S. adult population has current infection, with at least double this prevalence among “baby-boomers” born between 1945-1965^9^. As of 2008, only half of HCV-infected Americans in this age cohort were aware of their infection^15^. Despite recommendations issued in 2012 to perform routine testing for HCV for baby-boomers^16^ and in 2020 for all adults^17^, less than 20% of eligible U.S. adults have received testing^18^. Moreover, there are disparities by race as African Americans have: 1) higher HCV prevalence among baby-boomers; 2) lower access to HCV testing;^19^ and 3) lower access to curative treatments^20^.

Developing an appropriate response to incidentally discovered viral infection results involves ethical principles of beneficence, autonomy, and duty to rescue^21^. Since effective treatment is available for HCV, the principle of beneficence suggests that return of results is appropriate. The principle of autonomy suggests that participants should be able to decide whether they want to receive these results, but the potential health implications for these study participants as well as for other individuals with whom they interact could mitigate adherence to this principle. There is potential for harm from disclosure and potential for harm from non-disclosure of viral results. Harms of disclosure could include impact on study participants (anxiety and distress about findings) and on non-study participants (anxiety about disease risk, potential disease transmission from study participants, and knowledge of disease transmission to study participants). In addition, HCV is associated with stigma^22^. Although most genetic results are likely not immediately impactful enough to warrant a duty to rescue^21^, highly pathogenic infections could fall into that category.

Returning non-CLIA research results to study participants entrains substantial legal challenges. A National Academies of Sciences, Engineering, and Medicine report^23^ outlined three potential approaches to return research results to study participants: a) Performing testing in a CLIA laboratory (which would involve a CLIA chain of custody for the biospecimens); b) Ascertain that while the research results are obtained in a non-CLIA laboratory the process conforms to a proposed quality management system for research laboratories; and c) Return the non-CLIA results with appropriate caveats to study participants with IRB approval (if there is high enough likelihood of value to the study participant relative to the risks of harm). The first option was not viable for COPDGene, since the biospecimens were not collected in a CLIA chain of custody, and the proposed quality management system for research laboratories does not yet exist, negating the second option. Additionally, Evans and Wolf argued that an unsigned 2014 document posted on the Centers for Medicare and Medicaid Services (CMS) website should not be construed to limit return of research results to those from CLIA laboratories. Instead, investigators could offer non-CLIA results with a clear warning that they should not be used for diagnosis or treatment^24^. Thus, we elected to pursue the third option, and we obtained IRB approval from the 17 participating clinical centers to return HCV results to COPDGene participants.

Only a small minority of COPDGene participants reacted negatively to receiving research-based HCV results. Although several participants were frightened by the potential health risks of HCV, the vast majority (93.5%) of the participants in the HCV Follow-Up Study supported returning these results. Unsurprisingly, newly diagnosed participants were more likely to be pleased to learn about a potentially curable infection than previously diagnosed participants. In retrospect, the timeline for HCV detection should have been described more accurately when the study participants were initially informed about HCV results, which would have allayed concerns regarding delays in returning these results.

Limitations of the study include the relatively small percentage of participants that participated in the HCV Follow-Up Study. Although 30/31 participants (97%) in the HCV Follow-Up Study either reported a previous or received a new HCV diagnosis, the false-positive and false-negative rates for the research HCV RNA-seq results for predicting clinically confirmed HCV are uncertain since the HCV status of participants who did not participate in the HCV Follow-Up Study was not systematically ascertained. We did not perform clinical HCV testing on previously collected COPDGene samples for several reasons: 1) The study consent form did not specify that HCV clinical testing would be performed; 2) We believed that the available evidence from our previously published article on viral sequence detection suggested that the HCV RNA-seq results were likely at least reasonably accurate^14^; and 3) Since the samples were not handled with a CLIA chain of custody, clinical testing would be required to confirm the results. However, the high percentage of participants who spontaneously volunteered a previous HCV diagnosis at the initial phone call suggests that the research HCV RNA-seq test is likely at least reasonably accurate for clinical HCV. The small number of newly diagnosed HCV subjects limited assessment of participant reactions to obtaining these results through a research study. We focused on HCV return of results in this project, but future work could include return of other known and emergent viral infections as well. Future investigations should consider the impact of pathogenicity, transmissibility, and treatability of pathogens identified from research Omics assays on decision-making regarding return of research results.

In conclusion, HCV results identified incidentally during transcriptomic research studies can be effectively returned to research study participants with a carefully designed process. Investigators performing Omics assays in human biospecimens that may discover non-human transcripts indicative of common and treatable infections such as HCV should consider how to manage incidental results prospectively rather than retroactively. Proactive planning should include mentioning these possibilities in the consent form and discussions with the institutional review board before the onset of the study to establish approaches for returning incidental results.

## Methods

### Study population

COPDGene is an ongoing observational study of current and former smokers with and without COPD, including a smaller number (*n* = 454) of non-smoking controls; the total number of enrolled participants across 21 clinical centers in the United States was 10,718^13^. By design, two-thirds of COPDGene study participants were non-Hispanic White, and one-third were non-Hispanic Black. RNA-seq has been obtained on peripheral blood samples from the five-year follow-up visit, and microbial transcripts were assessed on 3,984 COPDGene participants.

COPDGene study participants provided written informed consent for the study protocol, which was approved at 21 clinical centers. Return of the HCV results and the HCV Follow-Up Study were approved by the 17 clinical center IRBs with HCV subjects identified (Baylor College of Medicine, Brigham and Women’s Hospital, Columbia University Medical Center, Reliant Medical Group, Lundquist Institute for Biomedical Innovation at Harbor-UCLA Medical Center, Minnesota Health Partners, Johns Hopkins University Medical Center, Morehouse School of Medicine, VA Ann Arbor Healthcare System, National Jewish Health, University of Pittsburgh, Temple University, University of Texas Health Sciences Center at San Antonio, University of Alabama at Birmingham, University of Iowa, University of Michigan, and University of California San Diego). Since there were no subjects with research evidence of HCV at the other four COPDGene clinical centers, IRB approval to return HCV results was not sought from those clinical centers.

Prior return of results in COPDGene has included alpha-1 antitrypsin deficiency (a well-documented autosomal recessive genetic risk factor for COPD and liver disease), spirometry results, chest CT findings, complete blood count abnormalities, and abnormal HADS (Hospital Anxiety and Depression Scale questionnaire) scores; these items were specifically included in the COPDGene informed consent form. For alpha-1 antitrypsin deficiency, the COPDGene consent form indicated that alpha-1 antitrypsin analysis would be performed in a research rather than a clinical laboratory, and that any reported research results would need to be confirmed through clinical testing. Participants were then asked: “Would you like to be informed about any abnormal alpha-1 antitrypsin results?” The COPDGene enrollment visit (2008-2012) template consent form also included the following text regarding other genetic results: “The test results from this study are not known to have any clinical significance at this time, and we will not tell you or any other individual about your specific genetic results.” No specific language was included in the COPDGene consent form regarding return of gene expression results. However, the 5-year follow-up visit COPDGene consent form (when the blood samples for RNA-seq that generated the viral infection results were collected) specified broad use of COPDGene blood samples: “Research using your samples and whole genome information is important for the study of virtually all diseases and conditions. Therefore, the data repositories will provide study data for researchers working on lung and other diseases.” However, the consent form did not include any language regarding return of these results to participants.

COPDGene has maintained contact with as many study participants as possible through semi-annual Longitudinal Follow-Up Program contacts (by e-mail, automated phone call, or coordinator phone call)^25^.

### Developing a return of results process

In response to the incidental identification of HCV results in COPDGene, a committee of COPDGene investigators and experts in bioethics and infectious diseases was established to determine whether to return HCV results and, if so, to develop a process for returning these results to COPDGene participants (committee members were co-authors EKS, AYK, BJM, EAR, JDM, JO, and PO). A group of nine COPDGene study participants was enlisted to join a COPDGene Participant Advisory Panel, and the procedures for returning HCV results were reviewed with these volunteers. These study participants provided their perspectives regarding whether HCV results should be provided to COPDGene subjects.

### Informing participants of hepatitis C virus

As recently reported^14^, 228 COPDGene participants with suggestive evidence of HCV (defined as “HCV results”) were identified by applying PathSeq^26^ to blood RNA-seq reads that were not mapped to the human genome. As shown in Table 1, we developed a procedure for returning HCV results, which separated the clinically important notification about potential HCV infection from a follow-up research study regarding the downstream effects of the return of results. This return of results procedure involved an initial contact letter (sent by Certified Mail) that specifically notified participants about their HCV research testing. This was in contrast to the commonly used procedure of providing a general contact letter that indicated an abnormal result had been found in their research samples and then giving them the option to learn about the specific finding. This decision was based on the individual and public health importance of HCV infection, as well as guidance from the COPDGene Participant Advisory Panel. We also included a phone contact by the participant’s COPDGene clinical center after the initial mailing (with a phone script) to explain the HCV results and assess their interest in receiving information regarding the HCV Follow-up Study. Participants were referred to their healthcare providers to determine if clinical HCV testing was appropriate. If participants needed a healthcare provider, the COPDGene clinical centers assisted in finding one. Although the phone script for this first call did not explicitly ask about previous diagnosis of HCV, many of these participants volunteered that they had previously been diagnosed with HCV, and this information was recorded.

A second mailing and phone call included the consent for the HCV Follow-up Study that involved notification of their primary care physicians, participation in a phone questionnaire approximately three months after their initial notification about HCV, and access to medical records relevant to HCV. The medical record review included prior HCV testing, HCV treatment, liver imaging, liver function testing, and evidence of liver disease.

The COPDGene Participant Advisory Panel endorsed this approach for returning HCV results to COPDGene participants. In addition, the COPDGene OSMB and IRBs for the 17 COPDGene clinical centers with at least one HCV-positive participant approved the return of results procedures. We elected to notify only participants with evidence for HCV infection (since the significance of a negative result is uncertain). We also decided to notify only surviving participants with evidence of HCV, and not to contact relatives of deceased participants.

### Assessing participant perceptions about HCV return of results

The HCV Follow-Up Study Questionnaire (Supplemental Materials) was developed to capture information regarding previous HCV diagnosis and treatment as well as the reactions of COPDGene participants to receiving HCV results. The HCV Follow-Up Study Questionnaire was IRB-approved at all relevant clinical centers, and written informed consent was obtained for participation in the HCV Follow-Up Study. Interested participants were asked to respond to this questionnaire by phone interview three months after receiving their initial contact letter regarding HCV results. Differences in reactions to receiving HCV results based on whether or not there was a previous HCV diagnosis were assessed with two-sided Fisher’s Exact Tests using SAS version 9.4.

### Supplementary information

Supplemental Materials

## Data Availability

COPDGene RNA-seq and phenotype data are available at dbGaP through accession number phs000179. The HCV Follow-Up Study Questionnaire is included in the Supplemental Materials.

## Code availability

RedCap was used for data collection. SAS Version 9.4 was used for statistical analysis. Analysis programs are available from the authors upon request.
