# Supplementary material for: Returning incidentally discovered Hepatitis C RNA-seq results to COPDGene study participants
Source: NPJ Genom Med. 2023 Oct 31;8:36. doi: 10.1038/s41525-023-00379-4 (PMC10616181; doi:10.1038/s41525-023-00379-4)
Supplement: Supplementary file 1 — Supplemental Materials [file 41525_2023_379_MOESM1_ESM.docx]

Supplemental Materials: Questionnaire for Participants in COPDGene Hepatitis C Return of Results Follow-Up Study

Question 1: Before being contacted by the COPDGene study about the Hepatitis C research results from your COPDGene blood samples, had you ever previously been diagnosed with Hepatitis C? YES NO

Question 2: After being contacted by COPDGene about your Hepatitis C research results, did you have a clinical blood test for Hepatitis C? YES NO

If YES, what was the result of the clinical Hepatitis C blood test?

1. It showed that I had evidence for Hepatitis C infection (positive test)
2. It showed that I did not have evidence for Hepatitis C infection (negative test)
3. I don’t know the results

Question 3: Have you received treatment with a medication for Hepatitis C? YES NO

If YES, when was this treatment given:__________

If YES, what was the name of the medication that you received?_________

Note that commonly used medications for Hepatitis C include:

Injections three times per week of interferon, along with ribavirin pills

Injections once per week of interferon, along with ribavirin pills

Harvoni (ledipasvir/sofosbuvir) pills

Viekira pak (paritaprevir/ritonavir/ombitasivir + dasabuvir)

Zepatier (elbasvir/grazoprevir)

Epclusa (sofosbuvir/velpatasvir)

Mavyret (glecaprevir/pibrentasvir)

The next set of questions relate to your reactions to receiving Hepatitis C research information from the COPDGene Study:

Question 4: The COPDGene Study did not anticipate returning information about Hepatitis C information when the study was designed. Do you think it was appropriate to return this information to you?

1. Yes
2. No

Question 4a: Please explain your response to Question 4:_____________________________

Question 5: The COPDGene Study provided Hepatitis C information to you by letter. Which approach would you have preferred for receiving this information?

1. Letter to me (which was done by COPDGene)
2. Phone call to me
3. Letter to my primary care physician, who could have spoken to me
4. Letter to me AND my primary care physician

Question 6: Please rate the information content provided in the initial letter that you received from the COPDGene Study regarding your Hepatitis C research results:

1. Very Informative
2. Informative
3. Neutral
4. Confusing
5. Very Confusing

Question 7: Please indicate which of the following responses captures your reaction to receiving the Hepatitis C information letter from COPDGene? (Circle all that apply)

1. I was frightened by the health risks of Hepatitis C
2. I was pleased to learn that a potentially curable infection was found
3. I was pleased that the study team informed me of this issue
4. I was upset that the study team contacted me about a result that I did not expect
5. Other (please describe): __________

Question 8: Please rate the information content in your discussion with COPDGene clinical center staff regarding your Hepatitis C research results:

1. Very Informative
2. Informative
3. Neutral
4. Confusing
5. Very Confusing

Question 9: How could the process of returning results to COPDGene participants about possible Hepatitis C infections based on research results be improved?

___________________________________________________________
